# Is there a role for ischaemic conditioning in cardiac surgery?

**DOI:** 10.12688/f1000research.10963.1

**Published:** 2017-04-25

**Authors:** Luciano Candilio, Derek Hausenloy

**Affiliations:** 1 The Hatter Cardiovascular Institute, Institute of Cardiovascular Science, University College London, London, UK; 2 The National Institute of Health Research–University College London Hospitals Biomedical Research Centre, London, UK; 3 Barts Heart Centre, St Bartholomew's Hospital, London, UK; 4 Cardiovascular and Metabolic Disorders Program, Duke-National University of Singapore Medical School, Singapore, Singapore; 5 National Heart Research Institute Singapore, National Heart Centre, Singapore, Singapore; 6 Yong Loo Lin School of Medicine, National University Singapore, Singapore, Singapore

**Keywords:** ischemic preconditioning, ischemic postconditioning, remote ischemic conditioning

## Abstract

Coronary artery disease (CAD) is a major cause of morbidity and mortality worldwide. Coronary artery bypass graft (CABG) surgery is the revascularisation strategy of choice in patients with diabetes mellitus and complex CAD. Owing to a number of factors, including the ageing population, the increased complexity of CAD being treated, concomitant valve and aortic surgery, and multiple comorbidities, higher-risk patients are being operated on, the result of which is an increased risk of sustaining perioperative myocardial injury (PMI) and poorer clinical outcomes. As such, new treatment strategies are required to protect the heart against PMI and improve clinical outcomes following cardiac surgery. In this regard, the heart can be endogenously protected from PMI by subjecting the myocardium to one or more brief cycles of ischaemia and reperfusion, a strategy called “ischaemic conditioning”. However, this requires an intervention applied directly to the heart, which may be challenging to apply in the clinical setting. In this regard, the strategy of remote ischaemic conditioning (RIC) may be more attractive, as it allows the endogenous cardioprotective strategy to be applied away from the heart to the arm or leg by simply inflating and deflating a cuff on the upper arm or thigh to induce one or more brief cycles of ischaemia and reperfusion (termed “limb RIC”). Although a number of small clinical studies have demonstrated less PMI with limb RIC following cardiac surgery, three recently published large multicentre randomised clinical trials found no beneficial effects on short-term or long-term clinical outcomes, questioning the role of limb RIC in the setting of cardiac surgery. In this article, we review ischaemic conditioning as a therapeutic strategy for endogenous cardioprotection in patients undergoing cardiac surgery and discuss the potential reasons for the failure of limb RIC to improve clinical outcomes in this setting. Crucially, limb RIC still has the therapeutic potential to protect the heart in other clinical settings, such as acute myocardial infarction, and it may also protect other organs against acute ischaemia/reperfusion injury (such as the brain, kidney, and liver).

## Introduction

Coronary artery disease (CAD) remains a leading cause of morbidity and mortality worldwide. For patients with complex multi-vessel CAD, coronary artery bypass graft (CABG) surgery is the revascularisation strategy of choice, as it offers survival advantage when compared to multi-vessel percutaneous coronary intervention (PCI) ^1, 2^. Although advances in surgical and cardioprotection techniques have resulted in improved clinical outcomes following CABG surgery, changes in patient demographics have meant that higher-risk patients are now undergoing CABG surgery, with an increased risk of perioperative myocardial injury (PMI), which is detected by the release of serum cardiac biomarkers such as CK-MB, troponin I, and troponin T, and a higher operative mortality risk of 5–6% ^3^. These changes include (a) the ageing population (the proportion of patients over 75 years old has increased by more than 4.5-fold over the last decade with a 5-year mortality in this age group of 35%), (b) the presence of co-morbidities such as diabetes and hypertension (the proportion of diabetic patients has risen from 15% to 22%, with an operative mortality of 2.6%), (c) more complex CAD is being operated on, and (d) concomitant valve and aortic surgery. Therefore, new treatment strategies are required to protect the heart from PMI during cardiac surgery in order to improve clinical outcomes in these higher-risk patients ^4, 5^. In this regard, the endogenous cardioprotective phenomenon of ischaemic conditioning has been investigated as a treatment strategy for protecting the heart and improving clinical outcomes in patients undergoing cardiac surgery.

## Ischaemic conditioning: evolution of an endogenous cardioprotective strategy

The myocardium possesses an innate ability to protect itself from the detrimental effects of acute ischaemia/reperfusion injury (IRI). This can be harnessed by subjecting the heart to one or more non-lethal cycles of brief (5–10 minutes) ischaemia and reperfusion, a phenomenon that has been termed “ischaemic preconditioning” (IPC) ^6– 8^. The concept of IPC was first discovered in a seminal study by Murry *et al*. in 1986 ^6^, when they made the surprising observation that four 5-minute episodes of regional myocardial ischaemia and reperfusion could dramatically reduce myocardial infarct (MI) size following a lethal period of ischaemia. IPC has since been reported to exist in every species and organ tested ^9^. The IPC stimulus elicits two windows of cardioprotection: the first one (termed “classical IPC”) begins immediately following the IPC stimulus and lasts for 2–3 hours ^6^, and the second one (termed the “second window of protection” or SWOP and first described in 1993 ^10, 11^) appears 12–24 hours after the IPC stimulus and lasts for 48–72 hours. The mechanisms underlying classical IPC have been extensively investigated, are complex, and involve the activation of plasma membrane receptors (such as adenosine, opioids, acetylcholine, catecholamines, angiotensin II, bradykinin, and endothelin), the recruitment of a number of signal transduction pathways (such as nitric oxide-PKG, reperfusion injury salvage kinase ^12– 14^, and survivor activator factor enhancement ^15– 18^), the inhibition of mitochondrial permeability transition pore (MPTP) opening ^19– 25^, and the prevention of necrotic and apoptotic cell death. The delayed cardioprotective effect of the SWOP has been shown to be mediated by the transcription of several new proteins such as inducible nitric oxide synthase, heat shock proteins, and cyclo-oxygenase-2 ^26^.

One major disadvantage of IPC is the need to apply the stimulus prior to the index ischaemic insult, which is not possible in acute myocardial infarction (AMI). In this regard, Zhao *et al*. in 2003 ^27^ found that applying three 30-second cycles of ischaemia and reperfusion to the canine heart at the onset of reperfusion following a period of index ischaemia reduced MI size to a level on a par with IPC, a phenomenon that was termed “ischaemic postconditioning” (IPost) and that has provided a therapeutic strategy to protect the heart following AMI ^28^. The signalling pathways underlying IPost are similar to classical IPC, although there are some differences ^7, 9, 29– 31^.

Crucially, both IPC and IPost require an invasive stimulus to be applied directly to the heart, thereby limiting their clinical application. In 1993, Pryzklenk *et al*. ^32^ made the intriguing discovery that applying the IPC stimulus (four 5-minute cycles of ischaemia and reperfusion) to the circumflex coronary artery could reduce MI size following a sustained occlusion of the left anterior descending coronary artery, demonstrating that the protection elicited by ischaemic conditioning could be transferred from one region of the heart to another, a phenomenon which has been termed “remote ischaemic conditioning” (RIC) ^32– 35^. Subsequent experimental studies demonstrated that the heart could be protected against AMI by applying the IPC stimulus to an organ or tissue remote from the heart, extending the concept of RIC to inter-organ ischaemic conditioning. The discovery that RIC could be induced by applying one or more cycles of brief ischaemia and reperfusion to the hind limb to reduce MI size ^36, 37^ facilitated the translation of RIC into the clinical setting with the use of a blood pressure cuff placed on the upper arm or thigh to induce one or more cycles of brief ischaemia and reperfusion to the limb (termed “limb RIC”) ^38^. The mechanisms underlying limb RIC are not known, especially those conveying the cardioprotective signal from the limb to the heart. The current paradigm suggests that the limb RIC stimulus generates a blood-borne transferrable factor, which then activates protective signal transduction pathways common to IPC and IPost, but the identity of the factor or factors remains unknown ^39^. Several potential candidates have been proposed, including nitrite ^40^, miRNA144 ^41^, and SDF ^42^, but conclusive evidence for their role as the mediators of RIC is lacking. Interestingly, the neural pathway to the limb has to be intact for RIC to be effective ^43, 44^, suggesting that the underlying factor or factors may be a neurotransmitter or neuropeptide.

## Ischaemic preconditioning and postconditioning in cardiac surgery

The first study to translate IPC into the clinical setting was by Yellon *et al*. in 1993 ^45^; they demonstrated that subjecting the heart to two 3-minute cycles of global ischaemia and reperfusion by clamping and unclamping the aorta was able to preserve myocardial ATP levels ^45^ and reduce PMI ^46^ following cardiac surgery. Since this pioneering study, a number of clinical studies have confirmed the cardioprotective effect of direct IPC in patients undergoing cardiac surgery, and a subsequent meta-analysis found that IPC was able to reduce ventricular arrhythmias, lower inotrope use, and shorten intensive care unit stay when compared to control ^47^. In 2007, Luo *et al*. ^48^ were the first to apply IPost to the setting of cardiac surgery when they showed that applying IPost at the time of aortic unclamping, by re-clamping the aorta after 30 seconds and then unclamping it for 30 seconds, a cycle that was repeated twice, reduced PMI in children undergoing cardiac surgery for Tetralogy of Fallot. A number of clinical studies have confirmed the efficacy of this IPost protocol in children and adults undergoing cardiac surgery ^49, 50^. Given the invasive nature of the IPC and IPost protocols and the risk of thrombo-embolism from serial clamping and unclamping of the aorta, neither IPC nor IPost has been applied in the clinical setting.

## Limb remote ischaemic conditioning in cardiac surgery

The first clinical trial to investigate limb RIC as a cardioprotective intervention in the setting of cardiac surgery was a small study of only eight patients by Günaydin *et al*. in 2000 ^51^. They found that limb RIC, comprising two cycles of 3-minute arm ischaemia and 2-minute arm reperfusion, did not reduce PMI during cardiac surgery. In 2002, Kharbanda *et al*. ^38^ characterised the use of a blood pressure cuff to non-invasively deliver limb RIC (three 5-minute cycles of ischaemia and reperfusion), demonstrating MI size reduction in a porcine model of acute myocardial IRI and improved endothelial function in human volunteers. The first clinical study to report a cardioprotective effect with limb RIC (three 5-minute cycles of arm ischaemia and reperfusion) was by Cheung *et al*. ^52^, who found less PMI in children undergoing corrective cardiac surgery for congenital heart disease. Our group was the first to demonstrate less PMI (43% reduction in serum troponin T release over a 72-hour postoperative period) in adult patients undergoing CABG surgery with limb RIC (three 5-minute cycles of arm ischaemia and reperfusion) when compared to control ^53^. Since these early studies, there have been a number of small positive studies confirming the cardioprotective effect of limb RIC in the setting of cardiac surgery, although there have also been several neutral studies (for comprehensive reviews, see 8, 54– 58). In a follow-up study of 329 CABG patients, Thielmann *et al*. ^59^ found that limb RIC (three 5-minute cycles of arm ischaemia and reperfusion) reduced PMI and actually reduced all-cause mortality at 1.5 years by 73% when compared to control. However, this study was not prospectively designed or powered to test the effects of limb RIC on major clinical outcomes following cardiac surgery.

The effect of limb RIC on clinical outcomes following cardiac surgery has been recently investigated in three large prospective multicentre randomised controlled clinical trials, all of which failed to demonstrate any benefit with limb RIC on either PMI or major clinical outcomes. The first of these was a South Korean clinical study of 1,280 patients undergoing cardiac surgery (CABG, valve, congenital heart disease, and aortic surgery), published in 2014 by Hong *et al*. ^60^. They found in adult patients that limb RIC (four 5-minute cycles of ischaemia and reperfusion administered twice to the upper limb before and after cardiopulmonary bypass) failed to improve the large primary composite endpoint (in-patient major adverse outcomes, including death, MI, arrhythmia, stroke, coma, renal failure or dysfunction, respiratory failure, cardiogenic shock, gastrointestinal complications, and multi-organ failure). The German RIPHeart clinical trial did not find any improvement in the in-patient primary composite endpoint (death, non-fatal MI, stroke, and acute kidney injury) with limb RIC (four 5-minute cycles of arm ischaemia and reperfusion) in 1,385 adult patients undergoing cardiac surgery (CABG, valve, and aortic) ^61^. Finally, the UK ERICCA clinical trial randomised 1,612 higher-risk adult patients undergoing CABG with or without valve surgery (Additive Euroscore ≥5) to either limb RIC (four 5-minute cycles of arm ischaemia and reperfusion) or control and failed to find any improvement in the 1-year primary composite endpoint (cardiac death, non-fatal MI, stroke, and coronary revascularisation) ^62^.

## Why did limb remote ischaemic preconditioning fail to improve clinical outcomes following cardiac surgery?

 The potential reasons why the three large clinical trials failed to find any reduction in PMI or improvement in short-term and long-term clinical outcomes following cardiac surgery with limb RIC include the following:

### 1. The clinical setting

CABG surgery may not be the optimum clinical setting to test the cardioprotective effects of limb RIC given that the extent of acute myocardial injury sustained in this clinical setting is relatively small and the fact that cardioprotection has been optimised by improvements in surgical and anaesthetic techniques and the use of myocardial preservation strategies such as hypothermia and cardioplegia ^63^. Moreover, patients undergoing concomitant valve surgery may be less amenable to RIC cardioprotection when compared to CABG surgery alone owing to the larger surgical trauma. Furthermore, RIC has been demonstrated in experimental studies to protect the heart mainly against acute IRI, whereas during cardiac surgery the causes of myocardial injury are multi-factorial and include inflammation (from cardiopulmonary bypass), direct handling of the heart, and coronary micro-embolisation. As such, limb RIC may be more likely to be effective in the setting of AMI, in which the target for cardioprotection is greater. In this regard, several clinical studies have reported a reduction in MI size with limb RIC applied prior to either thrombolysis ^64^ or primary PCI ^65– 71^ in ST-elevation MI (STEMI) patients, and a large European multicentre randomised controlled clinical trial (the CONDI2/ERIC-PPCI trial) is underway investigating whether limb RIC can reduce cardiac death and hospitalisation for heart failure at 1 year ^72^.

### 2. The limb remote ischaemic preconditioning protocol

Both the ERICCA and RIPHeart studies used a limb RIC protocol comprising four cycles of arm ischaemia and reperfusion ^61, 62^. Whether this is the optimal limb RIC protocol for cardioprotection in the setting of cardiac surgery is not known, as the RIC protocol has not been fully characterised in either the experimental animal or the clinical setting of acute myocardial IRI. Further work is therefore needed to investigate the most effective limb RIC (this has recently been done in mice ^73^), a task which would be made easier if a biomarker could be discovered, which can be used to assess the cardioprotective efficacy of limb RIC. However, this will be difficult given that the mechanisms underlying limb RIC remain unclear. It has also been suggested that the failure to fully blind the limb RIC protocol may have contributed to the positive results of the smaller clinical studies ^74^. Achieving full blinding of the limb RIC protocol in the setting of cardiac surgery can be challenging but is possible using a cuff attached to a dummy arm beneath the surgical drape ^75, 76^.

### 3. Experimental animal models

The majority of experimental studies demonstrating cardioprotection with limb RIC have used an experimental animal model of MI based on external occlusion of a coronary artery and have not tested limb RIC using a more relevant experimental animal model of cardiopulmonary bypass ^77^. Furthermore, the experimental studies have for the most part included healthy, juvenile, small and large animals, making them far removed from the clinical setting of the typical middle-aged patient with IHD and multiple co-morbidities and co-medications (see below) ^77, 78^.

### 4. Co-morbidities

A number of co-morbidities (such as age, diabetes, hypertension, and hypercholesterolaemia) have been shown in experimental animal studies to attenuate the cardioprotection induced by IPC and IPost, and emerging data suggest that limb RIC is also susceptible to this phenomenon ^77– 80^. Although some experimental studies have been able to recapitulate one individual co-morbidity (using diabetic, hypertensive, or hypercholesterolaemic animal models) when assessing cardioprotection, most patients have multiple co-morbidities and, furthermore, they are often on multiple treatments for their co-morbidities (anti-diabetic, anti-hypertensive, and lipid-lowering medication)—reproducing this in animal models will be extremely challenging ^58^.

### 5. Co-medications

Patients undergoing CABG surgery receive a number of different medications, many of which have the potential to interfere with the cardioprotection elicited by limb RIC—these include anti-diabetic medications, statins, angiotensin-converting enzyme inhibitors or angiotensin receptor blockers, calcium antagonists, beta-blockers, nitrates, morphine, inhaled anaesthetics, and propofol ^78^. Of these, it has been suggested that the use of the intravenous anaesthetic propofol may have contributed to the failure of limb RIC to reduce PMI and improve clinical outcomes in the ERICCA and RIPHeart studies, given that over 90% of patients received propofol ^61, 62^; however, the data supporting this proposition are not conclusive. The first clinical study to draw attention to the potential confounding role of propofol on limb RIC was by Kottenberg *et al*. ^81^, who showed that limb RIC was cardioprotective in the presence of isoflurane anaesthesia (n=19 patients), but not propofol anaesthesia (n=14 patients) in the setting of CABG surgery. Interestingly, propofol anaesthesia in the absence of limb RIC had no cardioprotective effect, suggesting that propofol was somehow antagonising the cardioprotective effect of limb RIC in the setting of cardiac surgery. More recently, Bautin *et al*. ^82^ showed in 48 patients (12 per group) undergoing aortic valve replacement surgery that the cardioprotective effect of limb RIC observed with sevoflurane anaesthesia was absent in the presence of propofol. In contrast, there have been several clinical studies reporting cardioprotection with limb RIC in cardiac surgery patients in the presence of propofol anaesthesia ^53, 83, 84^. Furthermore, there are experimental data suggesting that propofol itself can reduce MI size ^85^ and is cardioprotective in a porcine model of cardiopulmonary bypass ^86^ through anti-oxidant and mito-protective mechanisms. Therefore, a suitably powered prospective randomised controlled clinical trial is required to test whether propofol anaesthesia antagonises the cardioprotective effect of limb RIC in the setting of cardiac surgery when compared to volatile anaesthesia.

## Conclusions

Ischaemic conditioning has been investigated as an endogenous cardioprotective strategy for protecting the myocardium against PMI and improving clinical outcomes following cardiac surgery. Of these, IPC and IPost have been reported to reduce PMI, but, as they require direct application of the cardioprotective stimulus to the heart and because of the potential thrombo-embolic risk from repetitive clamping of the aorta, their clinical application has been limited. In this regard, limb RIC, which allows the cardioprotective stimulus to be applied to the arm or leg by simply inflating a blood pressure cuff placed on the upper arm or thigh, has facilitated RIC’s use in the clinical setting of cardiac surgery, where it has been shown to reduce PMI. However, three large multicentre clinical studies have failed to find improved short-term and long-term clinical outcomes with limb RIC following cardiac surgery, questioning the role of limb RIC in the setting of cardiac surgery. Further studies are required to investigate why limb RIC failed to improve clinical outcomes in this clinical setting. However, limb RIC still has therapeutic potential to protect the heart in AMI patients and may also protect non-cardiac organs (such as the brain, liver, and kidney) from acute IRI.
